# Transient bilateral cataract during intensive glucose control: a case report

**DOI:** 10.1186/s13256-017-1268-5

**Published:** 2017-04-12

**Authors:** Jung Hyun Park

**Affiliations:** Departmemt of Ophthalmology, Seoul Paik Hospital, Inje University College of Medicine, Marennae-ro 9, Jung-gu, Seoul, 04451 South Korea

**Keywords:** Diabetes, Cataract, Glucose control, Hyperopic shift, Osmotic stress, Case report

## Abstract

**Background:**

Cataracts are generally known to occur in hyperglycemic conditions in diabetic patients. In this case, cataract occurred in the course of glucose level control in a patient who had been in a hyperglycemic state.

**Case presentation:**

A 42-year-old Korean man who had uncontrolled diabetes for more than a year presented with bilateral posterior subcapsular cataracts, which developed within days of initiating antihyperglycemic therapy. With control of his serum glucose level for several weeks, the cataracts regressed.

**Conclusions:**

Transient cataracts can develop during the hypoglycemic state; prompt surgery should be deferred.

## Background

Cataracts are one of the earliest complications of diabetes mellitus (DM). Patients with diabetes mellitus are two to five times more likely to develop cataracts compared to nondiabetic patients [1].

The pathogenesis of cataracts in the hyperglycemic environment is well established [2]. There are some case reports regarding the development of transient diabetic cataracts while the patients were in a hyperglycemic state, which regressed after good glycemic control [3].

We describe a case of bilateral transient cataracts that developed while the patient’s blood glucose level was being rapidly lowered. Supportive photo-documentation was obtained.

## Case presentation

A 42-year-old Korean man visited a cardiology clinic for management of hypertension. He had been prescribed antihypertensive and lipid-lowering agents for years and had been followed up irregularly. The medications include losartan 50 mg, fenofibrate 160 mg and a combination capsule of simvastatin 20 mg and ezetimibe 10 mg. On routine checkup, his blood test results were as follows: fasting plasma glucose (FPG), 555 mg/dL; serum creatinine, 2.29 mg/dL; serum sodium, 140 mmol/L; serum triglycerides, 792 mg/dL; glycosylated hemoglobin (HbA1c), 14.9%. Review of his medical history revealed that he had high FPG levels 1 year prior to the consult during a routine checkup. Treatment had been recommended, but he did not undergo further workup. Our patient was immediately started on oral hypoglycemic agents and was referred to the department of ophthalmology for evaluation of diabetic retinopathy.

On ophthalmic examination, his uncorrected visual acuity (UCVA) was 20/200 and best corrected visual acuity (BCVA) was 20/20 in both eyes. He had mild myopia, with -3.25 diopters (D) of spherical equivalent in both eyes, which was the same as his own glasses. The lenses were clear in both eyes and the remainder of the ophthalmic examination findings were normal.

**Fig. 1:**
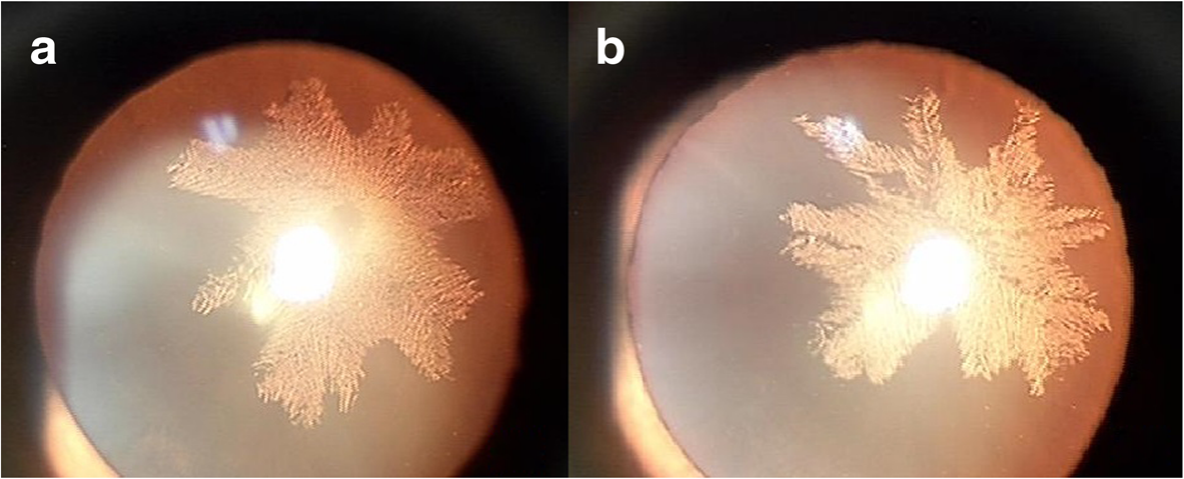
Posterior subcapsular cataract in the right (**a**) and left (**b**) eye which developed 3 days after starting glucose control

Three days after the first visit, our patient presented with a sudden decrease in visual acuity and glare in both eyes that had begun 2 days prior to consult. Metformin hydrochloride (Glycomin tablet) 500 mg twice a day was prescribed. However, his blood glucose level was not optimally controlled, so he was prescribed insulin with titration. On the day he was treated with insulin and his blood glucose level began to be controlled, he felt an abrupt decrease in his visual acuity. His BCVA was 20/40 in the right eye and 20/50 in the left eye. A dilated slit-lamp examination showed fine feathery snowflake opacities in the posterior subcapsular region of bilateral lenses (Fig. 1). Dilated fundus examination revealed some exudates and cotton wool patches from the macula to the mid-periphery region. Repeated blood test results on the same day showed a rapid decrease in FPG, which decreased to 214 mg/dL. His serum sodium level was 139 mmol/L and serum creatinine and triglyceride levels were reduced to 1.91 mg/dL and 424 mg/dL, respectively.

Ten days after the first visit, he complained that he could not see well with his own glasses. His visual acuity with his own glasses was 20/40 in the right eye and 20/50 in the left eye. His BCVAs were 20/25 in both eyes. Manifest refraction showed mild hyperopia, with +1.0 D of spherical equivalent in both eyes. A slit-lamp examination revealed essentially clear lenses except for scattered fine opacities in the posterior subcapsular region.

**Fig. 2:**
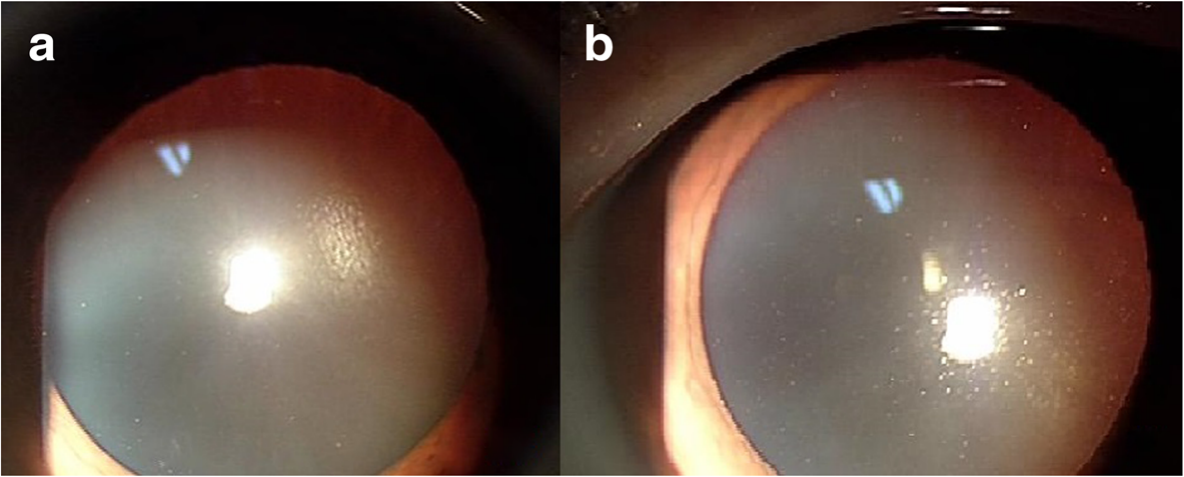
Regression of the posterior subcapsular cataract 4 weeks after initial presentation in the right (**a**) and left (**b**) eye

Four weeks after the first visit, his BCVA was 20/20 in both eyes. Manifest refraction was -1.25 spherical diopter (Dsph) -0.75 cylindrical diopter (Dcyl) × 30 in the right eye and -1.25 Dsph -0.75Dcyl × 110 in the left eye. On slit-lamp examination, the subcapsular opacity of the lenses had regressed, showing nearly clear lenses (Fig. 2). A fundus examination showed decreased exudates and cotton wool patches.

The antihypertensive and lipid-lowering medications that our patient had taken for several years were not changed during these clinical courses (Table 1).

**Table 1:** Timeline of our patient’s clinical course

Dates	Past medical history	
	High blood glucose level 1 year before initial visit	
Date	Symptoms and medical findings	Ophthalmic findings
Day 1	Fasting plasma glucose: 555 mg/dL	Best corrected visual acuity: 20/20 in both eyes
	HbA1c: 14.9%	Refractive error: -3.25 diopters (D) in both eyes
	Begins oral hypoglycemic agent	Clear lenses
		Diabetic retinopathy (-)
Day 2	Feels blurred vision	
	Switches to insulin	
Day 4	Fasting plasma glucose: 214 mg/dL	Best corrected visual acuity: 20/40 in the right eye; 20/50 in the left eye
		Bilateral subcapsular cataract
		Retinal hemorrhage and exudates
Day 11	Cannot see well with his own glasses	Visual acuity with his glasses: 20/40 in the right eye; 20/50 in the left eye Best corrected visual acuity: 20/25 in both eyes
		Refractive error: +1.0D in both eyes
Day 29	Better visual acuity	Best corrected visual acuity: 20/25
		Refractive error: -1.75D in both eyes
		Clearer lens

*D* diopters

## Discussion

Previous reports suggest that transient cataracts develop in hyperglycemic conditions, especially in type I DM, as sorbitol accumulates in lens fibers and regresses as blood glucose level is regulated [4]. However, this patient would have been hyperglycemic for a considerable period because he had been told that his blood glucose level was high 1 year prior to consult and his HbA1c result was 14.9%. As he had visited our clinic for routine screening evaluation before he was started on a hypoglycemic agent, his lenses were found to be clear on the first visit. Although we did not take a photograph of his lenses, his visual acuity was 20/20 in both eyes and he did not complain of blurred vision. Therefore, it can be postulated that the cataracts developed while the blood glucose level lowered sharply. The mechanism cannot be explained clearly, but it can be assumed that the homogeneity of the lens fiber is broken by the change in the membrane permeability because of the rapid osmotic pressure change [5].

At the same time, a hyperopic shift occurred temporarily. As the osmolality in aqueous humor was sharply lowered, the fluid would influx into the lens; the lens was swollen, which caused temporary hyperopia.

## Conclusions

Our case illustrates a possible effect of glycemic control. Transient cataracts can occur while the blood glucose level is being lowered from a hyperglycemic condition. This type of cataract can be completely reversible and refractive change can accompany it. Recognition of the potential reversibility of this type of cataract can avoid unnecessary surgery.
